# A deep network-based model of hippocampal memory functions under normal and Alzheimer’s disease conditions

**DOI:** 10.3389/fncir.2023.1092933

**Published:** 2023-06-21

**Authors:** Tamizharasan Kanagamani, V. Srinivasa Chakravarthy, Balaraman Ravindran, Ramshekhar N. Menon

**Affiliations:** ^1^Laboratory for Computational Neuroscience, Department of Biotechnology, Bhupat and Jyoti Mehta School of Biosciences, Indian Institute of Technology Madras, Chennai, TN, India; ^2^Department of Computer Science and Engineering, Robert Bosch Centre for Data Science and AI, Indian Institute of Technology Madras, Chennai, TN, India; ^3^Cognition and Behavioural Neurology Section, Department of Neurology, Sree Chitra Tirunal Institute for Medical Sciences and Technology, Trivandrum, Kerala, India

**Keywords:** associative memory recall, hippocampus, familiarity, dopamine, autoencoder, Alzheimer’s disease, picture-naming task, pattern completion

## Abstract

We present a deep network-based model of the associative memory functions of the hippocampus. The proposed network architecture has two key modules: (1) an autoencoder module which represents the forward and backward projections of the cortico-hippocampal projections and (2) a module that computes familiarity of the stimulus and implements hill-climbing over the familiarity which represents the dynamics of the loops within the hippocampus. The proposed network is used in two simulation studies. In the first part of the study, the network is used to simulate image pattern completion by autoassociation under normal conditions. In the second part of the study, the proposed network is extended to a heteroassociative memory and is used to simulate picture naming task in normal and Alzheimer’s disease (AD) conditions. The network is trained on pictures and names of digits from 0 to 9. The encoder layer of the network is partly damaged to simulate AD conditions. As in case of AD patients, under moderate damage condition, the network recalls superordinate words (“odd” instead of “nine”). Under severe damage conditions, the network shows a null response (“I don’t know”). Neurobiological plausibility of the model is extensively discussed.

## 1. Introduction

There is a long line of studies that implicate the role of the hippocampus in declarative memory functions (Milner et al., 1968; Steinvorth et al., 2005; De Almeida et al., 2007). Damage to the hippocampal region is seen during the course of Alzheimer’s disease and the normal course of aging (Golomb et al., 1993). In order to serve its function as a memory unit, the hippocampus must have access to the raw material for memory, which is sensory information. A quick review of the anatomy of the hippocampus and its place vis a vis the cortex provides useful insights into the mechanisms of its memory functions.

As a subcortical circuit, the hippocampus receives widespread projections from cortical areas in the temporal, parietal, and frontal lobes via the entorhinal cortex (Hasselmo, 1999; Insausti et al., 2017). A majority of hippocampal afferents from the posterior brain come from higher-order sensory and association cortices, areas that are capable of generating abstract representations of sensory information (Bowman and Zeithamova, 2018). Here representation refers to the compressed lower-dimensional feature vectors of cortical input. Thus, sensory information spread out over large cortical areas is projected, first to parahippocampal and perirhinal cortices, and then to the entorhinal cortex (EC), which is the gateway to the hippocampus (Burwell and Amaral, 1998).

The hippocampal formation connects several neural fields like the Dentate gyrus (DG), CA3, CA1, and subiculum (Schultz and Engelhardt, 2014). Nearly all the neural fields in the hippocampus receive projections from the superficial layers of the Entorhinal Cortex (EC) (Gloveli et al., 1998; Hargreaves et al., 2005; Brun et al., 2008). ECs afferent connections are formed using one trisynaptic pathway and two monosynaptic pathways (Yeckel and Berger, 1990; Charpak et al., 1995). The trisynaptic pathway consists of the perforant pathway between the second layer of EC (EC II) to DG (Witter et al., 1989), the mossy fibers between DG and CA3 (Claiborne et al., 1986), and Schaffer collaterals between CA3 to CA1 (Kajiwara et al., 2008). The monosynaptic pathways are formed between the second layer of EC (EC II) to CA3 (Empson and Heinemann, 1995; Gloveli et al., 1998) and the third layer of EC (EC III) to CA1 via perforant pathways (Witter et al., 1989). CA3 has more recurrent connections compared to the other hippocampal regions (Amaral and Witter, 1989), a feature that prompted researchers to attribute to it a crucial role in pattern completion and memory storage. The fifth layer of EC (EC V) receives the afferent projections from CA1 directly and indirectly via the subiculum (Canto et al., 2012; O’Reilly et al., 2013). It is this fifth layer of EC that sends back projections to widespread cortical targets that provided the actual sensory inputs (Insausti et al., 1997).

To summarize, there are bidirectional projections between the sensory cortex (high dimensional) and the hippocampus (low dimensional) (Hasselmo, 1999; Insausti et al., 2017). The hippocampal formation comprises multiple loops and extensive recurrent connections (Yeckel and Berger, 1990; Charpak et al., 1995). The above structure performs various memory processes such as memory encoding, recall, consolidation, and replay. The projections from the cortex to the hippocampus supports the memory encoding process (Yassa and Stark, 2011). The backward projections from the hippocampus to the cortical regions support memory recall by reconstructing the cortical state from the hippocampal representation (Renart et al., 1999). The loops and the recurrent connections in the hippocampal formation supports the memory replay and consolidation processes (Rothschild et al., 2017; Ólafsdóttir et al., 2018). In the current study, we focus on modeling two memory processes: memory encoding using pattern separation and recall using pattern completion.

**FIGURE 1:**
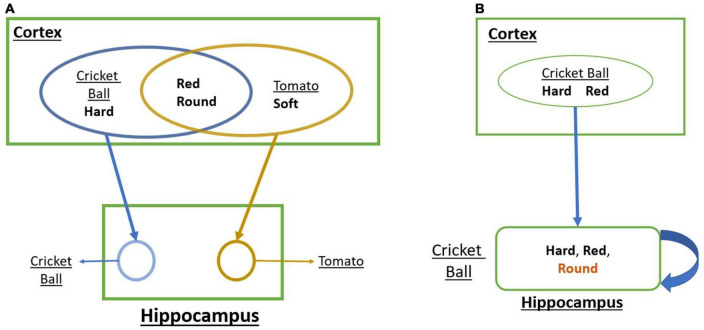
**(A)** A schematic that shows how convergent projections from the cortex to the hippocampus achieves Pattern Separation. **(B)** A schematic that shows how the loop dynamics over the cortical representations in the hippocampus achieves Pattern Completion.

The projection pattern from cortical areas to the hippocampus suggests that one of the prime features of the cortical state represented by the hippocampus is *pattern separation* (Yassa and Stark, 2011). Pattern separation refers to differentiating two or more patterns clearly even though they have several shared features. To illustrate the concept of pattern separation, let us consider the problem of representing a cricket ball vs. a tomato (Figure 1A). A cricket ball is round, red, and hard, while tomato is approximately round, red, and soft. Thus, the cortical representations of the two objects are likely to have a large overlap. But since the objects these feature combinations point to are quite distinct, it is desirable that the representations generated by the hippocampus are also adequately distinct, thereby achieving pattern separation.

There is another aspect of hippocampal memory function known as pattern completion (Mizumori et al., 1989; Rolls, 2013). Pattern completion refers to the reconstruction of a complete pattern from a partial or noisy pattern. Let us illustrate this concept using the same objects: a cricket ball and a tomato (Figure 1B). When we identify a cricket ball from a picture (red + round) even without touching it (hard), we are mentally supplying the missing feature of hardness. Similarly, a tomato can be identified visually without tactile exploration. These are examples of pattern completion that involve filling in missing features based on sensed features.

In order to understand how the hippocampus supports pattern separation and pattern completion, one must consider a crucial aspect of the anatomy of the hippocampus circuit. Since we will not be incorporating detailed hippocampal anatomy in the proposed model, we content ourselves with a simple schematic (Figure 1). One noteworthy feature is the presence of multiple loops with the hippocampus that take input from the superficial layers of EC and return the output to the deeper layers of EC, which send back projections to widespread cortical targets (Insausti et al., 1997).

A majority of hippocampal memory models involve implementations of pattern separation and pattern completion, distinguishing themselves in terms of anatomical details incorporated in the model or the specific memory tasks that they set out to explain (Marr, 1971; O’Reilly and McClelland, 1994; Rolls and Treves, 1994, 2012; McNaughton and Nadel, 2020). Gluck and Myers (1993) exploit the cortico-hippocampal projection pattern (Gluck and Myers, 1993), which they model as an autoencoder (Hinton and Salakhutdinov, 2006). An autoencoder is a special type of feedforward network where the network is trained to map the input onto itself (Hinton and Salakhutdinov, 2006). The autoencoder comprises two components: the encoder and the decoder. The encoder processes the input and generates a compressed, lower-dimensional representation of the input. This representation is sometimes called feature vector. The decoder uses the feature vector to reconstruct the input as expected. When the autoencoder is implemented with convolution layers, it is called a convolutional autoencoder. The nature of the autoencoder training ensures that, in this model, the hippocampus achieves pattern separation. The high-dimensional cortical state is the input to the autoencoder, while the hippocampus is the low-dimensional hidden layer. Thus, the cortico-hippocampal projections form the encoder, while the back projections representing the decoder are responsible for memory recall.

The pattern completion aspect of hippocampal memory function was highlighted by one of the earliest and most influential models of the hippocampus proposed by Marr (1971). Marr (1971) visualized a memory as a pattern distributed over a large number of neocortical neurons. Since the neocortical neurons have reentrant connections, it is possible to store patterns by association. Association refers to the establishment of a relationship between patterns. Activation of some neurons that represent a partial set of features can cause activation of neurons representing the remaining features, thereby achieving pattern completion. Mathematical associative memory models that exhibit pattern completion often involve networks with high recurrent connectivity and attractor dynamics (Hopfield, 1982, 1984; Amit, 1990). For example, Hopfield (1982) proposed a single-layered recurrent network that demonstrates attractor dynamics which has an associated energy function (Hopfield, 1982). Kosko (1988) proposed BAM (Bidirectional Associative memory), which is an extension of the Hopfield model on hetero-associative memory with similar attractor dynamics (Kosko, 1988). The high recurrent connectivity (4%) among the CA3 pyramidal neurons had inspired a long modeling tradition that treats CA3 as an associative memory (Amaral et al., 1990; De Almeida et al., 2007). Treves and Rolls (1994) have taken the associative memory view of CA3 and presented storage capacity calculations (Treves and Rolls, 1994). Wu et al. (1996) described the effect of noise of pattern storage in an associative memory model of CA3 (Wu et al., 1996). This has evolved a computational perspective that posits CA3 at the heart of pattern completion functions of the hippocampus.

There are other modeling approaches that describe pattern completion mechanisms of the hippocampus without specifically describing CA3 as an associative memory. The models of O’Reilly and McClelland (1994) and O’Reilly and Rudy (2001) describe the loop of connections from the superficial layers of EC, to DG to CA3 to CA1 back to deep layers of EC (Norman and Reilly, 2002), Hasselmo and Wyble (1997) present a model of hippocampal attractor dynamics that explains the disruptive effects of scopolamine on memory storage (Hasselmo et al., 1997). Thus, there is a spectrum of models that describe pattern completion functions of the hippocampus either by placing the burden of storage exclusively on CA3 and its recurrent connectivity or relying on the general internal loops of the hippocampus to supply the necessary attractor dynamics.

Similarly, Perlovsky (2001, 2007) and Perlovsky and Ilin (2012) proposed a new framework, neural modeling fields (NMF), which uses neural networks and fuzzy logic as a multi-level hetero-hierarchical system for modeling the mindClick or tap here to enter text. Here, perception has been modeled as the interaction between the bottom-up and top-down signals. Learning in this framework is driven by the dynamics, which increases the similarity value between the bottom-up signal (input signal for ex. visual stimuli), and the top-down signals (mental representations). The similarity is measured as the probability of the given input signal matching the representations of a particular object. In this approach, the input signal (bottom-up signal) is compared for similarity measure with multiple top-down signals. Here the top-down signals are generated from multiple simulators/models (running in parallel), each producing a set of prime representations for the objects expected. Thus the prime-representations (with the higher similarity measure) and their parameters are selected and used to fit with the bottom-up signals. With this process, the vague (noisy) bottom-up signal is transformed into a crisp signal through an iterative process, thus it demonstrates pattern completion behavior. The model by Perlovsky uses a set of predefined models for each object. Though the model recognizes the actual pattern from the noisy images, one model is maintained for each object.

The aforementioned review of computational models of hippocampal memory functions shows a common structure underlying a majority of the models embodying two crucial features: (1) They impose some form of autoencoder structure, with feedforward/feedback projections, on the cortico-hippocampal network, thereby achieving pattern separation and a compact representation of the cortical state. (2) They use the attractor dynamics arising, either solely within CA3 or, more broadly, in the hippocampal loops to achieve pattern completion. Instead of addressing the sensitive task of having to pick the best among the above models, we propose to construct a model with the above features but cast in the framework of deep networks so as to exploit the special advantages offered by deep networks.

Although often criticized for possessing inadequate biological plausibility, in recent years, deep networks have enjoyed surprising success in modeling the activities of visual, auditory, and somatosensory cortical hierarchies (O’Reilly and McClelland, 1994; O’Reilly and Rudy, 2001; Norman and Reilly, 2002; Kanitscheider and Fiete, 2017). For example, Deep networks trained on visual recognition tasks matched the error patterns from human across object classes (Cichy et al., 2016; Geirhos et al., 2018). Deep network models on Auditory domain such as speech and music recognition match human-performance (Hinton et al., 2012; Kell et al., 2018; Jang et al., 2019). Yamins et al. (2014) recapitulated the aspects of the ventral visual hierarchy using deep neural networks by relating intermediate layers to V4 and the later layers to the Inferior Temporal cortexClick or tap here to enter text. Kanitscheider and Fiete (2017) using a recurrent neural network demonstrated that the hidden representations of the network exhibited the key properties of hippocampal place cells in navigation problems. Various models using deep neural networks have also been employed in somatosensory systems (Zhuang et al., 2017), hippocampus, and EC (Kanitscheider and Fiete, 2017; Banino et al., 2018; Cueva and Wei, 2018). Some studies explain the learning characteristics of the hippocampus using an autoencoder structure (Benna and Fusi, 2021; Santos-Pata et al., 2021). A review by Ramezanian-Panahi et al. (2022) explained the need for this kind of abstract models for better interpretation of brain dynamicsClick or tap here to enter text. Although the interpretation of the inner layers in deep networks is hard at the level of individual neurons, these networks have well-defined structures at the level of layers. In the feedforward neural networks, the hierarchical organization of input from one particular layer to the next layer recapitulates the aspects of the hierarchical structure of the brain. Though some progress has been made in using deep networks for modeling hippocampal spatial navigation functions, modeling memory functions is still in its early stage (Kanitscheider and Fiete, 2017).

The concept of familiarity invariably figures in most discussions of the memory functions of the hippocampus. Studies on human memory that draw from cognitive, neuropsychological, and neuroimaging methodologies suggest that human memory is composed of two processes of memory: recollection and familiarity (Henson et al., 1999; Yonelinas, 2001; Yonelinas et al., 2005; Droege, 2017). Sometimes when we meet a person, we may simply have the sense that the person is familiar but not remember the person’s name or when and where we have first met that person. This sense of having met before refers to familiarity, while the ability to recall the various features that constitute that object refers to recollection. Many studies have established the link between the hippocampus and familiarity-based memory functions. Wixted (2004) showed that the hippocampus is crucial for representing familiarity (Wixted, 2004). Kirwan and Stark (2004) showed that the hippocampus is selectively activated during familiarity-based recollection tasks (Kirwan and Stark, 2004).

Another vital element for memory processing in the hippocampus is dopamine. A considerable body of neurobiological literature links dopaminergic signaling with reward processing (Wise and Rompre, 1989). Using classical conditioning experiments, Schultz et al. (1997) took a further step and demonstrated strong analogies between dopaminergic activity in Ventral Tegmental Area (VTA) and an informational signal known as temporal difference (TD) error in Reinforcement Learning (Schultz et al., 1997). This connection has inspired extensive computational modeling efforts that sought to connect dopaminergic signaling with the function of the basal ganglia (BG), an important subcortical circuit linked to dopamine signaling (Schultz et al., 1997; Chakravarthy et al., 2010; Chakravarthy and Moustafa, 2018).

Although dopamine signaling, in the context of the BG, is often associated with motor function, there is extensive evidence linking dopamine to cognition and memory functions (Goldman-Rakic, 1997; Kulisevsky, 2000; Koch et al., 2014; Martorana and Koch, 2014). Packard and White (1989) demonstrated memory enhancement on the application of dopamine agonists (Packard and White, 1989). Dopamine agonists, like Bromocriptine, enhanced memory performance in the elderly (Morcom et al., 2010). It is possible to find neuroanatomical evidence within the hippocampal circuitry in order to support the aforementioned studies that link memory deficits with dopamine. Although there was an early view that dopamine does not modulate hippocampal neural activity, subsequently, evidence was gathered for the existence of mesencephalic dopamine projections in rat hippocampus (Penfield and Milner, 1958; Gasbarri et al., 1994) and the influence of dopamine on hippocampal neural fields (Mansour et al., 1992; Hsu, 1996; Hamilton et al., 2010).

The importance of dopamine in novelty-based memory encoding and recall has been observed in many studies (Holden et al., 2006; Duszkiewicz et al., 2019). Some experimental studies have related dopamine release to learning novel stimuli (Bardo et al., 1993; Schultz, 1998). Few studies associated higher dopaminergic activity to novel stimuli and lower dopaminergic activity to familiar stimuli (Brown and Aggleton, 2001; Kamiński et al., 2018).

It is well-established that dopamine represents reward prediction error (Temporal Difference error) in reward-based decision-making (Schultz et al., 1997). In the novelty aspect, we relate dopamine to stimulus prediction error. The network would not have learnt any representation for any novel stimuli, leading to higher prediction errors. As the network learns the stimuli, the prediction error also reduces, which leads to lesser dopamine activity. This explains that the hippocampus represents some value function that encodes the familiarity information.

Here we have proposed familiarity as a notion that is complementary to novelty, and the hippocampal-VTA loop codes the familiarity value for the learned information. With this idea, we show that the cortico-hippocampal interactions maximize the familiarity function computed in the hippocampal circuit. The memorization process involves a gradual transition from novelty to familiarity. So, it is possible to assume that the goal is maximizing familiarity.

The hippocampus supports two kinds of associative memories: auto-associative memory and hetero-associative memory. The pattern completion is an example of auto-associative memory, where the input and the output are of same modality. The picture-naming task is a classic example of hetero-associative memory (Barbarotto et al., 1998; Cuetos et al., 2005). In this task, the participants are asked to name the pictures shown on the screen. This task is used to assess the level of cognitive deterioration in AD patients. AD is a progressive neurodegenerative disorder in which neuronal loss is observed throughout the brain. The initial loss of neurons is detected in the Entorhinal cortex and hippocampus (Gómez-Isla et al., 1996; Bobinski et al., 1998). Alzheimer’s patients at different stages show different kinds of responses in the picture-naming task. The controls and early stage AD patients predominantly produce correct responses (e.g., to the picture of a lion, they respond with the word “lion”). In the mild to moderate stage, they make some semantic errors (e.g., responding with words that are similar or closer to the actual word semantically–like tiger in place of lion–or the superordinate words–like animal instead of lion). In the severe stage, they predominantly make Semantic Errors or No Response (I don’t know) (Barbarotto et al., 1998; Cuetos et al., 2005).

In this paper, we present a deep network-based model of hippocampal memory functions. In this model, the feedforward and the feedback projections between the sensory cortex and the hippocampus are modeled as encoder and decoder of an autoencoder. The network receives images as input. The network has a deep autoencoder structure with the inner-most layer, the Central Layer (CL), representing the hippocampus–more specifically, CL can be compared best to Entorhinal Cortex (EC). Furthermore, attractor dynamics is imposed on the state of CL by assuming that the state of CL constantly seeks to find the local maximum of a *familiarity* function, where the familiarity refers to the confidence at which an object is remembered (Skinner and Fernandes, 2007). Once the state of CL with the maximum familiarity is achieved, the state is passed to the decoder for the reconstruction of the image. The model incorporates the two crucial features of hippocampal memory models–pattern separation and pattern completion. The convergent projections from the input layer to the Central Layer, which represent the Autoencoder, are thought to achieve pattern separation. The hill-climbing dynamics over the familiarity function computed in the Central Layer, which represents the hippocampus, is thought to achieve pattern completion. The proposed network exhibits more accurate recall performance than one without the attractor dynamics over the familiarity function. The proposed network exhibits more accurate recall performance than one without the attractor dynamics over the familiarity function. In general, autoencoder-based networks inherently removes the noise to some extent due to the generalization effect. When an autoencoder is trained to map noisy patterns to noiseless patterns, then better pattern completion can be observed. But if the autoencoder is trained to map the same noisy version of the input, then pattern completion performance is lesser, and this happens due to the generalization effect. But in the proposed model, we map the input to the same noisy version. This training process ensures that the autoencoder does not eradicate the noise in its representation. We show that familiarity value representation is needed for pattern completion in the proposed settings.

Going beyond the basic model, we implement the picture-naming task by introducing two pipelines in the network architecture–one for the image and another for text. We apply the resulting “picture-naming model” to simulate the performance of Alzheimer’s patients. When the hidden layer neurons are progressively destroyed, in order to simulate hippocampal damage in Alzheimer’s, the model’s recall performance showed a strong resemblance to the performance of the patients on the same task.

## 2. Methods and results

### 2.1. Auto-associative memory model

The model of auto-associative memory is explained using a modified convolutional autoencoder, in which the Central Layer is associated with attractor dynamics. We call such an architecture an Attractor-based Convolutional Autoencoder (ACA). The attractor dynamics arises out of performing hill-climbing over a cost function, which in the present case is the familiarity function. The performance of the proposed model is compared with a standard convolutional autoencoder and a recurrent convolutional autoencoder. All the three architectures are compared on image pattern completion tasks.

#### 2.1.1. Dataset

**FIGURE 2:**
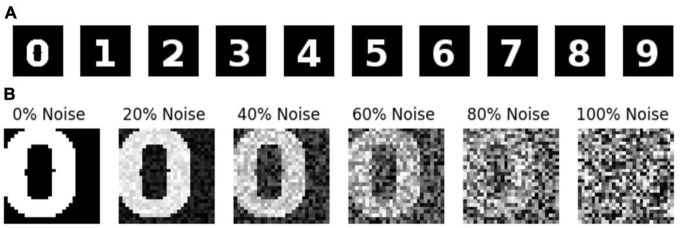
**(A)** Digit Images without noise. **(B)** Image of Zero at different noise levels.

The image dataset is generated using the images of printed numerals 0–9 (Figure 2A) with size 28 × 28. The dataset consists of images with various noise levels. The noisy images are generated using equation (1).


(1) $$I_{n}=|I-\eta .G|\in R^{28x28}$$

Here *I*, and *I_n_* denotes the noiseless Image, noisy Image, respectively. *G* represents the noise matrix, whose individual element is given as *G*_*ij*_ = *U*(0, 1). Where *U*(0, 1) is a uniform random variable with values ranging between 0 and 1. η is a scalar that specifies the noise percentage, which ranges between 0 and 1. The modulus operator is used to keep the image pixel values between 0 and 1. The noisy sample images are shown in Figure 2B. The training dataset contains 100,000 images, the validation dataset contains 20,000, and test dataset contains 20,000 images. The images are categorized into ten classes (0–9) depending on the source image it is generated.

#### 2.1.2. Familiarity value function

Here the assumption is that when a particular pattern is well learned, the reconstruction gets better. Even from a partial pattern or noisy pattern, it is quite possible to recall the complete familiar information. In the proposed model, familiarity is related to the correctness value that encodes the noise level in the input pattern (image). Thus, for a familiar pattern, the value will be maximum (1), and vice versa. The dataset contains an image and a corresponding familiarity value. Here the familiarity (correctness) value *C* is estimated using equation (2).


(2) $$C=e^{-\frac{{||I-I_{n}||}^{2}}{2\sigma^{2}}}$$

where *I_n_* denotes the noisy version of an image. *I* represents the perfect/noiseless Image. Here σ is set to 15. Whereas the value *C* for a noiseless image is 1, for a noisy image, the value ranges between 0 and 1. For each training image, the corresponding value is calculated using equation (2).

#### 2.1.3. Standard convolutional autoencoder (SCA)

**FIGURE 3:**
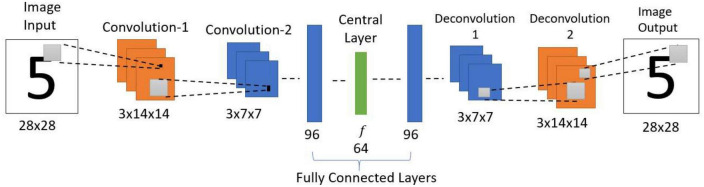
Architecture of standard convolutional autoencoder. This network is trained to reproduce the observed input image as the output. i.e., if there is a noise in the input, the network needs to learn the noise along with the input.

The standard convolutional autoencoder network (Figure 3) takes an image as input and maps it onto itself (i.e., learns the image along with the noise). In the present case, the encoder comprises two convolution layers with max-pooling followed by two fully connected layers. The decoder comprises two fully connected layers followed by two deconvolution layers, thereby producing the output of the same size as the input.

#### 2.1.4. Image encoder

The encoder uses the input image of dimension 28 × 28. It comprises two convolution layers, with each convolution layer extracting three feature maps over a receptive field of size 3 × 3. Each convolution layer is followed by a max-pooling layer (Scherer et al., 2010) of stride 2 generating feature maps, each of size 3 × 14 × 14 and 3 × 7 × 7, respectively. The output of the second convolution layer is flattened (3 × 7 × 7 to 147) and connected to a fully connected layer with 96 neurons. This, in turn, is connected to the Central Layer with 64 neurons. Here all the layers use the leaky-ReLU activation function (Maas et al., 2013), but the Central Layer uses the sigmoid activation function.

#### 2.1.5. Image decoder

The image decoder generates an image using the features from the Central Layer. Here, the final layer of the encoder is connected to a fully connected layer with 96 neurons. This, in turn, is connected to a fully connected layer with 147 neurons, which is then reshaped to 3 × 7 × 7. This reshaped data is fed to the first deconvolutional layer, which has three filters of size 3 × 3 with stride 2 and produces an output of size 3 × 14 × 14. Then the first deconvolutional output is fed to the second deconvolutional layer (3 filters of size 3 × 3 with stride 2) to produce the image output of size 28 × 28. Here the output layer uses a sigmoid activation function, and all the other layers use leaky-ReLU activation function.

#### 2.1.6. Recurrent convolutional autoencoder (RCA)

**FIGURE 4:**
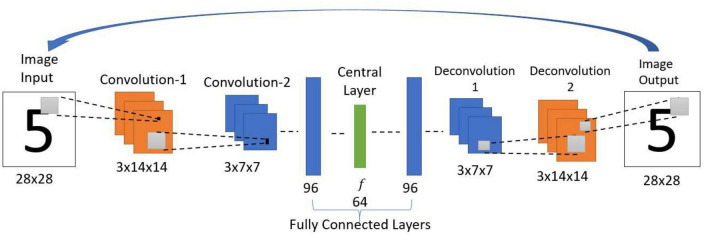
Architecture of recurrent convolutional autoencoder. This network structure and the parameters are the same as the Standard Convolutional Autoencoder. One difference is that the current iteration’s output is used as input in the next iteration. After multiple iterations, the settled pattern is used as output.

This model uses essentially the same architecture as the one in the standard convolutional autoencoder network above but with a difference: instead of decoding the input in one-step, the output of the network at the current iteration is used as input in the next iteration (Figure 4). Thereby forming a loop, the network acts as an attractor, and the stable output obtained after several iterations is considered as the final retrieved pattern.

#### 2.1.7. Attractor-based convolutional autoencoder (ACA)

**FIGURE 5:**
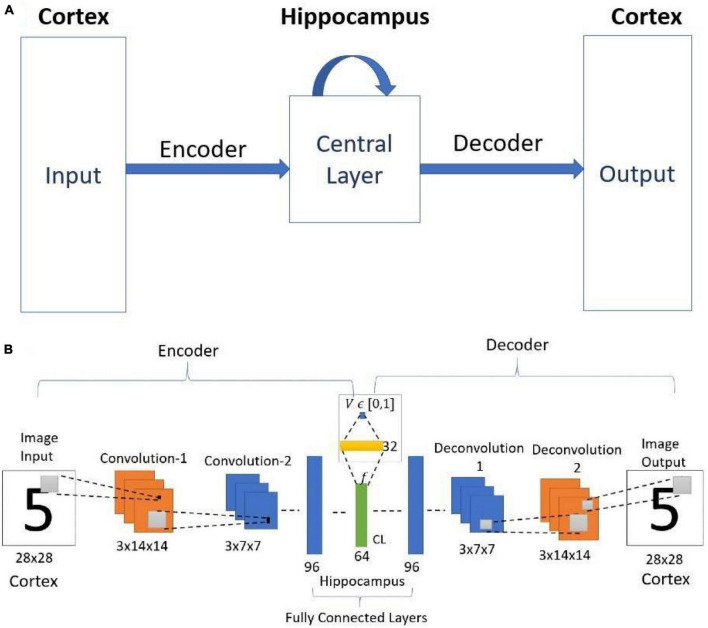
**(A)** Schematic diagram of the cortico-hippocampal memory Network. **(B)** Architecture of Value-based Convolutional Autoencoder Network. CL- Central Layer. Here the value function estimated from the encoder layer represents the familiarity (correctness value).

This model also uses the same network architecture as used in the simple convolutional autoencoder above, but with an important modification (Figure 5B). Figure 5A shows the schematic diagram of the cortico-hippocampal network. Here the architecture of the encoder and the decoder are the same as used in the simple convolutional autoencoder network. The network maps the input to itself. Therefore, during training, the network is trained to reproduce the noisy input as the output. The noisy images are generated using equation (1).

This model uses the concept of familiarity, and each input to the network is mapped to a scalar value that represents familiarity. In the network, the “familiarity unit” is implemented by a single sigmoidal neuron, which receives the inputs from the central layer (CL) with 64 neurons (attributed to EC) via a sigmoidal layer with 32 neurons. This unit outputs a scalar value V representing the familiarity level of the input. This single node predicts the familiarity (correctness) value *C* [equation (2)]. After training, the familiarity value predicted by the network is used to reach the nearest best feature with the maximum familiarity value.

#### 2.1.8. Training

##### 2.1.8.1. Standard convolutional autoencoder

Unlike the conventional denoising autoencoders, the standard convolutional autoencoder is trained to produce the same noisy input as output. This approach is imposed to imitate the natural conditions, as the brain doesn’t perceive noisy input and corresponding noiseless output. The standard autoencoder network is trained to minimize the cost function ℒ_*sca*_ (a combination of multiple cost functions) as given below.


(3) $${ℒ}_{1}={||I-\bar{I}||}^{2}$$

(4) $${ℒ}_{2}=-\sum_{i=0}^{9}\left({\text{p}}_{\text{i}}.\log\left(\bar{p_{i}}\right)+\left(1-{\text{p}}_{\text{i}}\right).\log\left(1-\bar{p_{i}}\right)\right)$$

(5) $${ℒ}_{sca}={ℒ}_{1}+\lambda_{1}*{ℒ}_{2}$$

Here,

*I*−input image.

$\bar{I}$-predicted Image.

p_i_- actual probability of input image being in i^th^ class.

$\bar{p_{i}}$- predicted probability of being in i^th^ class.

λ_1_−trade-off parameter.

ℒ_1_ denotes the image reconstruction error. ℒ_2_ denotes classification error (cross-entropy). SoftMax layer with ten neurons is used for classification over the Central Layer. The classification layer is used to make the encoded features separable for the image inputs of different numbers. The network parameters are updated using Adam optimizer (Kingma and Ba, 2015).

##### 2.1.8.2. Recurrent convolutional autoencoder (RCA)

As the standard convolutional autoencoder itself is merely used iteratively, there is no separate training employed in this case.

##### 2.1.8.3. Attractor-based convolutional autoencoder (ACA)

The Attractor-based convolutional autoencoder is trained similarly to the standard convolutional autoencoder along with an additional cost function for the familiarity value prediction. Here the attractor-based convolutional autoencoder is trained to minimize the cost function ℒ_*aca*_ [Equation (7)].


(6) $${ℒ}_{3}={||C-V||}^{2}$$

(7) $${ℒ}_{aca}={ℒ}_{sae}+\lambda_{2}*{ℒ}_{3}$$

Here,

*C* - desired familiarity value.

*V*−predicted familiarity value.

λ_2_−tradeoff parameter.

ℒ_*sae*_-the cost function used in the standard convolutional autoencoder.

ℒ_3_ denotes the familiarity value prediction error. Here also the network is trained using Adam optimizer.

In this network, for a given input image, the output is retrieved after modifying the encoded feature vector using the familiarity value. The feature vector is modified using the predicted familiarity value to attain the maximum familiarity value by the hill-climbing technique. Here Go-Explore-NoGo paradigm is used to implement the stochastic hill-climbing behavior.

#### 2.1.8. Go-Explore-NoGo (GEN)

Similar to Simulated Annealing (Kirkpatrick et al., 1983), the GEN algorithm allows us to perform hill-climbing over a cost function without explicitly calculating gradients. Although originally derived in the context of modeling the basal ganglia, it can be used as a general optimizing algorithm. The Go-Explore-NoGo (GEN) policy (Chakravarthy and Balasubramani, 2018), consists of 3 regimes: Go, Explore, and NoGo. A slightly modified version of GEN is used in this model. The Go regime decides that the previous action must be repeated. The NoGo regime forbids from taking any action. [There is another variation of the NoGo regime wherein the action taken is opposite of the corresponding action in the Go regime (Chakravarthy and Balasubramani, 2018)]. The Explore regime allows choice of a random action over the available action space. For a given input image, the feature vector *f* from the Central Layer and the corresponding familiarity value *V* is estimated. The network aims to identify the nearest feature vector with a maximum familiarity value of 1. It is achieved using the following algorithm based on the GEN policy (Chakravarthy and Balasubramani, 2018).

Let.

*f*- be the current feature vector for the given input image.

*V*- familiarity value for the feature vector *f*.

∈ -threshold value.

ϕ−is a 64-dimensional random vector, where each element ϕ_i_ is given as.

$$\phi_{\text{i}}=U\left(0,1\right)$$

Where *U*(0, 1) is a standard uniform distribution variable with values between 0 and 1.

Initialize △*f*(0)=0

f(t+1)=f(t)+△f(t)

δ(t)=V(t+1)-V(t)

*f* is updated by the following equations:


if δ(t)>∈:△f(t+1)=△f(t)    -``Go″else if δ(t)=-∈:△f(t+1)=-△f(t)  -``NoGo″(8)else△f=ϕ        -``Explore″End.

Thus, when the network receives a noisy version of the input image, the feature vector is extracted in the Central Layer of the network. The feature vector is modified iteratively using the above algorithm until the corresponding familiarity value reaches the maximum. Once the familiarity value attains 1 (or the local maximum), then the latest modified feature vector is given to the decoder, and the output image for the proposed model is produced.

#### 2.1.9. Performance comparison

The performance of the above three networks is compared on the pattern completion task for the same set of images.

For the standard convolutional autoencoder, the output image for the given input image is taken while no hill-climbing dynamics is applied over the encoded feature vector.

The recurrent autoencoder (Autoencoder with the outer loop, where the output for the current iteration is given as input for the next iteration) forms a loop, and therefore the network acts as an attractor. In this case too, there is no additional dynamics applied to the encoded feature vector, which is the output of the Central Layer. So, the output settles at a particular image output over multiple iterations for a given input image. This settled image pattern is taken as the final output of the network.

**FIGURE 6:**
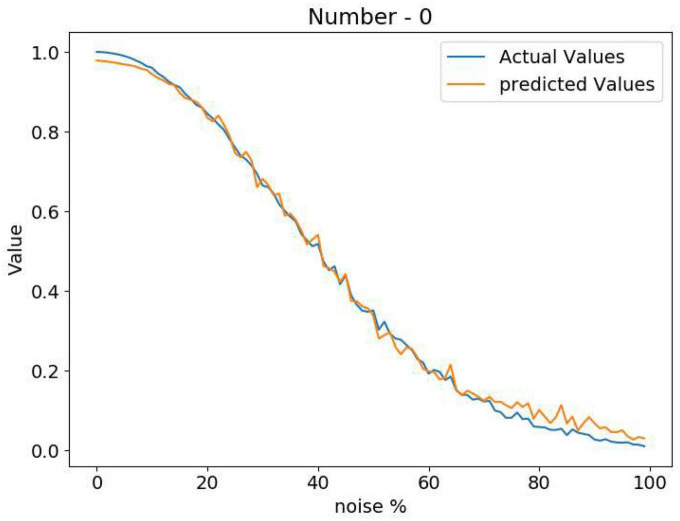
Comparison between the actual familiarity value and the network predicted value at different noise levels.

The Attractor-based Autoencoder model retrieves the output after applying the familiarity dynamics to the Central Layer output using the familiarity value. The familiarity value, V, is extracted from the single node using the feature vector from the Central Layer (Figure 5B). Figure 6 shows the familiarity value concerning the noise percentage for images of zero at different noise levels. Here the actual familiarity value is derived using equation (2). The predicted familiarity value is the output of the single value node.

#### 2.1.10. Results

**FIGURE 7:**
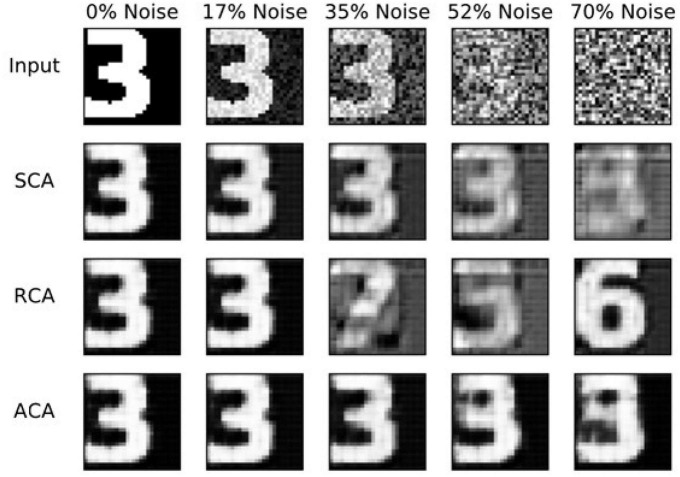
Image reconstruction comparison for Image three at different noise levels. SCA, standard convolutional autoencoder; RCA, recurrent convolutional autoencoder; ACA, attractor-based convolutional autoencoder.

The performance on the pattern-completion task is compared here for the above three models. Figure 7 shows the output comparison at different noise levels. The first row has the input images of “3” at five different noise levels. The second, third, and final rows show the outputs of the standard (SCA), recurrent (RCA), and the proposed Attractor-based convolutional autoencoder Model (ACA), respectively. It clearly shows that the network with the GEN technique outperforms both the other methods in reconstructing the proper images from the noisy images.

**FIGURE 8:**
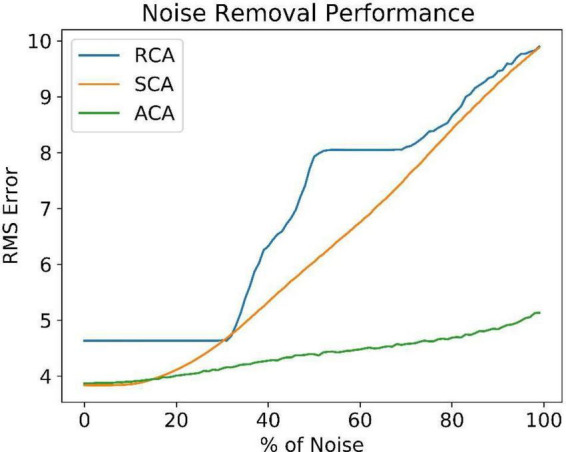
Comparison of reconstruction error between SCA, RCA, and ACA at different noise levels.

Figure 8 compares the noise reduction/removal capability (RMS error) among the three models. The RMS error is estimated using the equation (9).


(9) $$RMSerror=||Y-\bar{Y}||$$

where *Y* is the expected noiseless Image and $\bar{Y}$ is the network output. Even at higher noise levels, the ACA model retrieves better noiseless images. It explains the need for an inner loop that estimates and uses the familiarity function. The number of iterations required to reach the maximum familiarity value is included in the Supplementary Figure 4.

### 2.2. Hetero-associative memory model

The hetero-associative memory model is demonstrated using a multimodal autoencoder network (Ngiam et al., 2011). The network used here is an extension of the value-based convolutional autoencoder. The hetero-associative memory behavior is instantiated in the image-word association task, which can be compared to the behavior of AD patients in the picture-naming task.

#### 2.2.1. Multimodal autoencoder network

**FIGURE 9:**
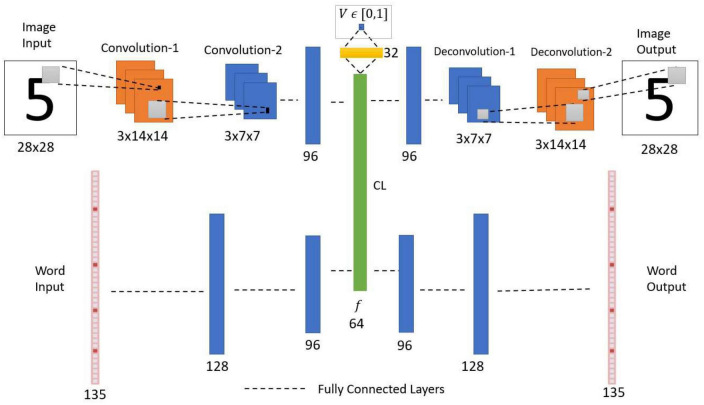
Network architecture of multimodal autoencoder with an associated value function. CL, central layer. The network receives two types of inputs (Image and Word). The CL establishes the association between the Image encoded feature vector and the Word encoded feature vector. The value function predicts the noise level in the input combination.

The multimodal autoencoder network has two components, the Image Autoencoder and the Word autoencoder. Both the components here are joined at a Central Layer (Figure 9). The Image Autoencoder and the Word autoencoder take images and words as inputs, respectively. A similar network configuration was used in another model called the *CorrNet* to produce common/joint representations (Chandar et al., 2016). The Image encoder uses two convolution layers with max-pooling followed by two fully connected layers. The Word encoder uses three fully connected layers. The outputs of the image encoder and word encoder are combined to make a common/joint representation. From this common representation layer, a single neuron is connected via a sigmoidal layer with 32 neurons, which outputs a scalar value representing the familiarity level of the input. The image decoder with two fully connected layers followed by two deconvolutional layers uses the joint representation to generate the image output. Similarly, the word decoder with three fully connected layers uses the joint representation to generate the Word output.

The robustness of associative memory behavior is tested by resetting the neurons at the Central Layer at different percentage levels for a given input image and generating the image and word outputs.

#### 2.2.2. Image encoder

Similar to the previous convolutional autoencoder network, the image encoder uses two convolution layers with the same number of filters of the same size. The second convolutional layer output is connected to a fully connected layer with 96 neurons. All the hidden layers use the leaky-ReLU activation function (Maas et al., 2013).

#### 2.2.3. Word encoder

The Word input is processed by the encoder with fully connected layers. The encoder takes a vector of size 135 as input. This vector represents five characters each by a 27 sized vector, thus 5 × 27 = 135. A detailed explanation of this vector is given in the dataset section. The input layer is connected to the first layer with 128 neurons. The first layer is connected to the second layer with 96 neurons.

#### 2.2.4. Joint representation

The outputs of both the image encoder and the word encoder are connected to a layer with 64 sigmoidal neurons. This generates a common feature vector. The common feature vector is estimated using the following equation (10).


(10) $$f=sigmoid\left(\frac{g_{I}.f_{PI}+g_{w}.f_{PW}}{g_{I}+g_{w}}\right)$$

where *f*_*PI*_,*f*_*PW*_ denotes the pre-feature vector (before applying activation function) from the Image encoder and the Word encoder, respectively; *g_I_* and *g_W_* are binary values representing the availability of image and word inputs, respectively, where 1 denotes the availability of a particular input, and 0 denotes the non-availability of input.

#### 2.2.5. Familiarity value function

The Central Layer is connected to a single sigmoidal neuron via a single hidden layer with 32 sigmoidal neurons. The single output sigmoidal neuron estimates a scalar value denoting the familiarity level of the input image-word combination.

#### 2.2.6. Image decoder

The Image decoder uses the same architecture as in the standard convolutional autoencoder network.

#### 2.2.7. Word decoder

The Word decoder takes the common feature vector as input and processes it through 3 fully connected layers that have 96, 128, and 135 neurons, respectively. The first two layers use the leaky-ReLU activation function. The output layer uses 5 SoftMax functions, out of 27 neurons each. Here each SoftMax function specifies one character.

#### 2.2.8. Dataset

The dataset used here is a combination of Images, Words, familiarity values, and association indices. The images are used from the dataset as in the autoencoder network.

#### 2.2.9. Words

The Word input is represented using five numbers of 27-dimensional one-hot vector representations, which together form a vector of size 135 (= 27 × 5). A single character is represented by a 27-dimensional vector. Among the 27 dimensions, the first 26 dimensions represent alphabets (a-z), and the last (27th) dimension represents the special character–empty space. For example, for the character “e,” the 5th element in the vector is set to 1, and the rest of the elements are set to 0. The number of characters is chosen to be five, considering the maximum number of characters in the words for numbers from zero to nine.

The Word input data is generated for the number-names (*zero*, *one*, *two*, …., *nine*) and the number-type-names (*even*, *odd*) (Table 1).

**TABLE 1:** Word inputs.

Number-names	
Zero	One
Two	Three
Four	Five
Six	Seven
Eight	Nine
**Number-type names**	
Even	Odd

The noisy words are generated using equation (11). This way, 12,000 noisy words are generated and used.


(11) $$W_{n}=|W-\eta .G|$$

Where *W*,*and W*_*n*_ denotes the proper and noisy Words, respectively. *G* is a noise vector with dimension 135, and each element of which is sampled from the standard uniform distribution *U*(0, 1).

#### 2.2.10. Familiarity value

The familiarity value for both the Image and Word is calculated using the Gaussian formula as in Equations (12, 13).


(12) $$V_{I}=e^{-\frac{{||I-I_{n}||}^{2}}{2.\sigma_{I}^{2}}}$$

(13) $$V_{W}=e^{-\frac{{||W-W_{n}||}^{2}}{2.\sigma_{W}^{2}}}$$

Where *I*, *W*, *I*_*n*_, *W*_*n*_ denotes the noiseless Image, noiseless Word, noisy Image, and noisy Word, respectively. Here σ_*I*_ is set to 50 and σ_*W*_ is set to 8. Thus, a noise-free image/word has a familiarity of 1; when there is noise, it will have a value between 0 and 1 depending on the level of noise.

When both the image and word inputs are presented to the network, the combined Familiarity is calculated by multiplying the familiarity value of the Image and the Word. This familiarity value is used by the network to reach the nearest best feature vector with maximum value by the Go-Explore-NoGo technique.

#### 2.2.11. Association index (γ)

The association index, γ, is a scalar that specifies the relation between the Image and the Word. For various combinations of the above images and words, the association index is generated. The rules followed for generating the association index are listed below.

• If a word denotes the same number-name or number-type-name for the given Image, then γ is set to +1.
• If a word does not match with either the number-name or number-type-name for the given Image, then γ is set to −1.

For example, “0” (Image) and “zero” (word) have an association index of +1, similarly “0,” and “even” also get the association index of +1. But, “0” and “odd” have an association index of −1. At a particular instant, at least one among the Image or word data should be present. When one modality among Image and Word is absent, the association value is set to 0.

The motivation here is not to learn the association index. The association index is used to establish the correlation among the feature vectors corresponding to the co-occurring images and words. Here the occurrence of image and the number-name (ex., “0”–“zero”) happens 80% of the time, and image and number-type-name (ex., “0”–“even”) happens 20% of the time. This ensures a higher correlation among the feature vectors of an image to the number-name than the correlation among the feature vectors of the image to the number-type-name.

Along with all the above input data, two more binary values *g_I_* and *g_W_* are also used, which represent the presence of the image input and the Word input, respectively.

#### 2.2.12. Training

For training the network, a combination of Image, Word, familiarity value, and the association index are used. The three input-output combinations used for training the network are shown in Table 2.

**TABLE 2:** Input-output combinations for the multimodal autoencoder.

Input	Output
Image, Word	Image, Word, Combined Familiarity Value, and Correlation-coefficient
Image	Image, Image Familiarity Value
Word	Word, Word Familiarity Value

The multimodal network is trained to produce the same given inputs as outputs irrespective of the noise in the input.

Different Image-Word combinations are used for training the network. The various combinations are as follows:

1. Noiseless Image, noiseless Word.
2. Noisy Image, noiseless Word.
3. Noiseless Image, noisy Word.

Here’ noisy image and noisy word’ combination is not used for training.

Among the above three combinations in the training dataset, each of the above combinations has 100, 100,000, and 12,000 data, respectively. Though each group has a non-uniform data count, while training, each batch contains 100, 1,000, and 120 combinations from the three groups, respectively. Since the noiseless image-noiseless word combinations are used across all the batches, and there is some overlap among these groups, it helps avoid overfitting the dataset with a larger size.

The network is trained to minimize the cost function ℒ [Equation (19)]. A combination of 5 cost functions ℒ_1_, ℒ_2_, ℒ_3_, ℒ_4_, ℒ_5_ are used for training. ℒ_1_, ℒ_2_, ℒ_3_, ℒ_5_ are image reconstruction error, word reconstruction error, the correlation coefficient of Image pre-feature vector and Word pre-feature vector, and value reconstruction error, respectively. ℒ_3_ is used to form the relationship between the feature vectors of the Image and the Word. ℒ_4_ is used to make the Image and Word feature representations closer.


(14) $${ℒ}_{1}={||I-\bar{I}||}^{2}$$

(15) $${ℒ}_{2}=-\sum_{i}\left(W_{i}.\log\left(\bar{W_{i}}\right)+\left(1-W_{i}\right).\log\left(1-\bar{W_{i}}\right)\right)$$

(16) $${ℒ}_{3}=\frac{\sum \left({\text{f}}_{PI}-\bar{{\text{f}}_{\text{PI}}}\right)\left({\text{f}}_{\text{Pw}}-\bar{{\text{f}}_{\text{Pw}}}\right)}{\sqrt{\Sigma {\left({\text{f}}_{\text{PI}}-\bar{{\text{f}}_{\text{PI}}}\right)}^{2}\Sigma {\left({\text{f}}_{\text{Pw}}-\bar{{\text{f}}_{\text{Pw}}}\right)}^{2}}}$$

(17) $${ℒ}_{4}={||{\text{f}}_{\text{PI}}-{\text{f}}_{\text{Pw}}||}^{2}$$

(18) $${ℒ}_{5}={||V-\bar{V}||}^{2}$$

(19) $$ℒ={ℒ}_{1}+\lambda_{1}*{ℒ}_{2}-\lambda_{2}*\gamma *{ℒ}_{3}+\lambda_{3}*{ℒ}_{4}+\lambda_{4}*{ℒ}_{5}$$

Where,

I–input image.

W–input word.

$\bar{I}$-predicted Image.

$\bar{W}$-predicted Word.

f_PI_-the pre-feature vector for Image.

f_Pw_- the pre-feature vector for the Word.

$\bar{{\text{f}}_{\text{PI}}}$- the mean pre-feature vector for Image.

$\bar{{\text{f}}_{\text{Pw}}}$- the mean pre-feature vector for the Word.

λ_1_, λ_2_, λ_3_, λ_4_ - tradeoff parameters.

γ−association index.

V–desired familiarity value.

$\bar{V}-$predicted familiarity value.

Here, the mean of the pre-feature vectors is estimated for a particular batch of images and words, respectively. The network parameters are updated using Adam optimizer (Kingma and Ba, 2015). After training, the results are generated with various combinations of inputs.

#### 2.2.13. Results

**FIGURE 10:**
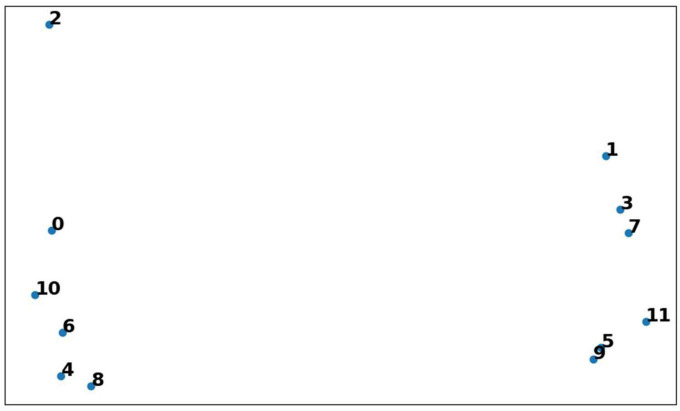
Vector representation of word features in 2D space. These features are generated by giving the Word inputs alone. Two clusters are formed for each category (even and odd). This explains the characteristic of pattern separation, where similar patterns form a cluster and non-similar patterns are far away in the feature space.

The results are generated by giving only one input (either Image or word) at a time. Figure 10 visualizes the common feature vector (size 64) in 2D space (using the two principal components with the highest portion of the total variance explained). The Word inputs are given to the network while keeping the image inputs blank (zero values). A total of 0–9 specifies words’ zero,’ “one,”…, “nine,” respectively, where “10” corresponds to the word “even,” and “11” corresponds to the word “odd.” Note that the words corresponding to the odd type and even type form separate clusters.

**FIGURE 11:**
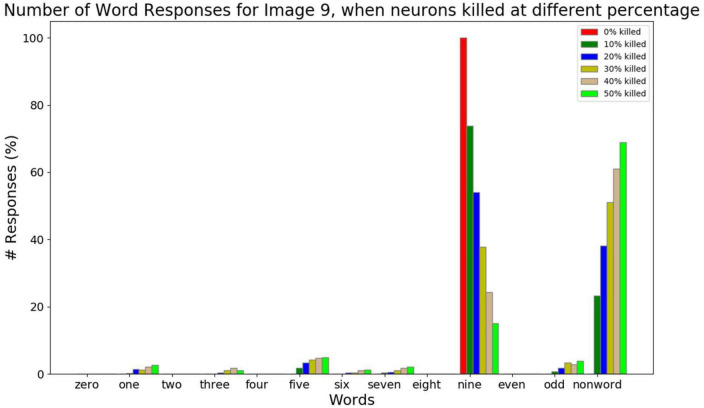
The response counts for all the number-names and the number-type-names while resetting different percent of neurons (0, 10, 20, 30, 40, 50, and 60%) for the image input of number 9. Here neuronal loss is related to resetting the neurons. Correct response (“nine”) is observed when there is no neuronal loss. For 10–30% neuronal loss, the responses belonging to the same category (“one,” “three,” “five,” “seven,” and “odd”) are observed, which is related to the semantic error. For 40–50% neuronal loss, most responses are non-word responses, which is attributed to no response.

**FIGURE 12:**
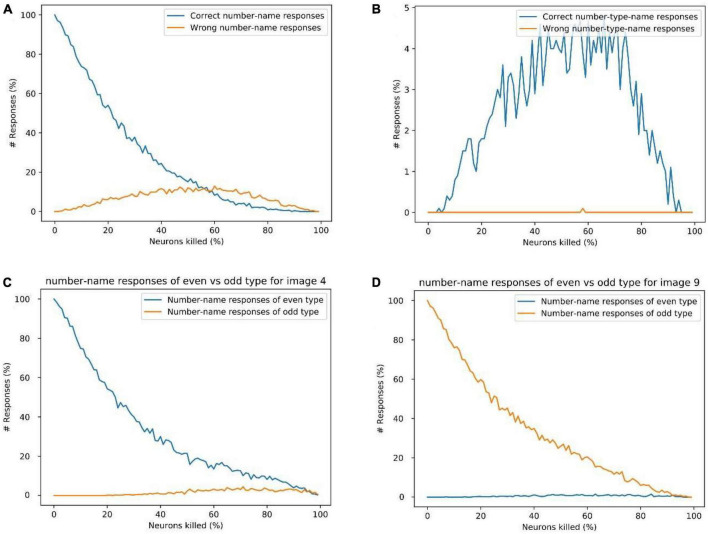
**(A)** Response percentage comparison of correct number-name (“nine”) vs. wrong number-names (“zero,” “one,”…,” eight”) for image input 9, **(B)** comparison of percentage of correct number-type-name (“odd”) vs. wrong number-type-name (“even”) responses for image input of “9.” **(C)** Sum of the count of even number-name responses vs. odd number-name responses for image input 4. **(D)** The sum of the count of even number-name responses vs. odd number-name responses for image input “9.” These results show that the possibility of a wrong number-name or wrong number-type response is minimal for a given image input, which explains the logic behind the observation of semantic errors.

The results below (Figures 11, 12) are generated by giving image input alone while keeping the Word input to be empty (zero values).

#### 2.2.14. Simulating the behavior of Alzheimer’s disease (AD) patients on picture-naming task

Alzheimer’s disease is characterized by the loss of cells in the Entorhinal cortex, a cortical area that serves as the gateway to the hippocampus (Gómez-Isla et al., 1996; Bobinski et al., 1998). Since the Central Layer, and the associated Familiarity computation network represent the Hippocampus in the proposed model, AD pathology is simulated by randomly killing/resetting a percentage of the neurons in the Central Layer. Then Go-Explore-NoGo policy is applied over this modified feature vector to reach the feature vector with maximum familiarity value. Note that the killed/reset neurons do not participate in the computation. The graphs below are generated by counting each word output’s responses at the Word decoder out of 1,000 times.

We consider three kinds of responses for a given image input while resetting neurons at the Central Layer. They are number-name responses (zero, one, …, nine), number-type-name-responses (even, odd), and non-name (nonword) responses (anything other than number-names and number-type-names).

The response percentage of all the number-names and number-type-names for the image input “9” is shown in Figure 11. In order to simulate AD pathology at different levels of degeneration, in the Central Layer, different percent of neurons (0, 10, 20, 30, 40, and 50%) are reset, and the average response count is calculated. From this, we can observe that as the percent of neurons being reset increases, the correct responses decrease. When the image input “9” is presented, and no Central Layer neuron is reset, the network produces the word “nine” all the time as expected. When 10–30% of neurons are reset, it produces the word “nine” most of the time. Among the wrong responses, most of them are either number-name responses or number-type-name responses of the same type/category (in this case: one, three…, nine, and odd). In other words, the responses of number-names (one, three, five, seven, and nine) and number-type-name (odd) of the same group are high compared to the number-names (zero, two, four, six, and eight) and number-type-name (even) of a different group. This can be related to the semantic error. When 40–50% neurons are reset, the sum of all the number-name and number-type-name responses falls below 30%, and the non-name response count is higher, which is similar to No-response.

Figure 12A shows the count of correct number-name response (“nine”) and wrong number-name responses (all the number-names except “nine”) while resetting different percent of neurons for the input of image “9.” This doesn’t include the number-type name responses such as “odd” and “even.”

Figure 12B shows the count of the correct number-type-name response (“odd”) and wrong number-type-name response (“even”) while resetting different percent of neurons for the input of image “9.” The number-name responses such as “zero,” “one,” etc., are not counted here.

From Figures 12A, B, We can observe that, as the percentage of neurons being reset increases, the response count of correct number-names (nine) reduces gradually, whereas the response count of the wrong number-names and the correct number-type-name (odd) increase gradually for some time and decrease after that. Here among the wrong number-name responses, most of them are of the same type but different number-name responses (one, three, five, and seven), which can be related to the semantic error.

Figures 12C, D show the count of even number-name responses vs. odd number-name responses (excluding the number-type-names) for the image input “4” and “9,” respectively. It can be observed that, for a given image input, the chance of producing a number-name word response of the wrong category is very small. This also explains the logic behind the occurrence of semantic errors. The analysis of different response types and Alzheimer’s patient’s behavior in picture-naming task is shown in the Supplementary Figures 1–3.

## 3. Discussion

We present a deep network-based model of the associative memory functions of the hippocampus. The cortico-hippocampal connections are abstracted out into two structural modules of the proposed model. In the first module, the bidirectional cortico-hippocampal projections are modeled as an autoencoder network. In this second module, the loop of connections from EC into the hippocampal complex and back to EC is modeled as hill-climbing dynamics over a *familiarity* function.

In the first part of the study, the model is used to simulate auto-associative memory functions using pattern completion task under normal conditions. The pattern-completion task is modeled using a convolutional autoencoder with an associated familiarity function. The autoencoder’s encoder and decoder are related to the feedforward and feedback projections between the sensory cortices and the hippocampal formation. There are many conventional denoising autoencoders proposed to solve this problem (Tian et al., 2019). These models use a supervised learning approach, where the noisy patterns are mapped to noiseless patterns during training. This kind of mapping does not fit the actual scenario, where the brain is not always presented with noisy and noise-free versions of the same pattern. The present study maps the noisy patterns to the same noisy version itself. The model learns to construct a noise-free version on exposure to a large sample of noisy patterns.

The Standard Convolutional Autoencoder does not have any attractor dynamics since the output is retrieved just in one step. The Recurrent Convolutional Autoencoder (RCA) shows attractor dynamics due to the loop created between the output and input layers. There is no explicit energy function handled in this RCA model. In the Attractor-based Convolutional Autoencoder (ACA), we combine Reinforcement Learning and Attractor Dynamics in an interesting way. We show that the Value (Familiarity) function actually serves the role of an explicit energy function, the hill-climbing over which generates the required attractor dynamics. It uses GEN-based attractor dynamics for hill-climbing, which is thought to be generated by the loop dynamics within the hippocampus. In RCA, when there is significant noise in the input image the network state can be kicked into a nearby attractor. Thus, it shows the wrong number image instead of the expected number image (Figure 7). As the noise level increases, it possibly jumps to an attractor corresponding to a different number. In SCA, as the output is retrieved directly from the input, the RMS error increases gradually. In ACA, the joint training of autoencoder along with the familiarity value function seems to create deep basins of attraction for each stored pattern, without minimum chance of creating spurious attractors.

### 3.1. Familiarity and the hippocampus

Several proposals were made regarding neuroanatomical substrates of familiarity and recollection. Based on the memory performance of patients with medial temporal lobe damage, some researchers suggested that while the hippocampal region is necessary for recollection, the surrounding cortical structures like the parahippocampal gyrus are essential for familiarity (Eichenbaum et al., 1994; Huimin et al., 1999). Another proposal links recollection with medial temporal lobe structures and familiarity with existing memory representations in the neocortex (Mandler and DeForest, 1979; Mandler, 1980; Graf and Mandler, 1984; Graf et al., 1984). Other proposals suggest that the hippocampus is important for both the familiarity and recollection processes (Manns et al., 2003; Malmberg et al., 2004; Wais et al., 2006). Wixted and Squire (2010) show that familiarity is supported by the hippocampus when the memories are strong (Wixted and Squire, 2010). They also argue that the hippocampus and the adjacent regions do not exclusively support only one process (Wixted and Squire, 2010). Although several authors accept the existence of dual processes–recollection and familiarity–there is no consensus on the neural substrates of recollection and familiarity. We now present a neurobiological interpretation of the computation of familiarity in the hippocampal circuitry. Mesencephalic dopaminergic signals have a major role to play in the proposed theory.

### 3.2. The role of dopamine in the memory functions of the hippocampus

Lisman and Grace (2005) presented an extensive review of experimental literature to establish dopaminergic projections to the hippocampus and show that dopaminergic neurons that project to the hippocampus fire in response to novel stimuli (Lisman and Grace, 2005). Electrophysiological recordings showed that dopamine cells in VTA, projecting to the hippocampus, increased their firing rate in response to novel stimuli; the activity of these neurons reduced with increasing familiarity (Steinfels et al., 1983; Ljungberg et al., 1992). Considering that novelty is the complementary notion to familiarity, the above body of evidence can be invoked to support our model that requires the computation of familiarity in the hippocampus. With the above background information, the central hypothesis of the proposed model may be expressed as follows: *the cortico-hippocampal interactions with regard to memory operations are based on maximizing familiarity computed within the hippocampal circuit. Since the process of memorizing a pattern entails a gradual transition from novelty to familiarity, this assumption of maximizing familiarity seems to be intuitively plausible.*

In the present study, the familiarity function [equation (2)] is trained by supervised learning that involves a direct comparison of the target pattern with the predicted pattern. It is also possible to train the familiarity function by Reinforcement Learning (RL) (Sutton and Barto, 2018), where a close match between the target and recalled pattern results in a reward. The familiarity function then, in mathematical terms, becomes the value function.

An RL-based formulation of hippocampal memory functions has an added advantage. The reward signal can be used not only to represent the level of match between the target and recalled pattern but also to represent the saliency of the pattern to the animal/subject. Several existing accounts that posit CA3 as the site of memory storage in the hippocampus, argue that the decision to store or not to store depends solely on the mismatch between the target and stored pattern (Treves and Rolls, 1992; Hasselmo et al., 1995). But a memory mechanism that stores all novel stimuli encountered by the animal in its interactions with the world, irrespective of the saliency of the stimuli to the animal, would glut the animal’s memory resources. The best possible way is to store only the important stimuli by filtering it based on the salience factors such as reward, novelty, recency, and emotional involvement (Cheng and Frank, 2008; Singer and Frank, 2009; McNamara et al., 2014; Santangelo, 2015). These notions will be explored in our future efforts.

### 3.3. Modeling hetero-associative memory function in Alzheimer’s disease (AD)

The second part of the work demonstrates hetero-associative memory using a multimodal autoencoder. In this case, the network is trained to form association between images and words at the Central Layer. Here the trained feature vectors belonging to the same category form a cluster (even and odd). AD patients’ behavioral response during the picture-naming task is reproduced by killing/resetting the neurons at the Central Layer.

In general, Alzheimer’s disease is linked to dysfunction in the cholinergic system. According to the cholinergic hypothesis, disruptions of the cholinergic system in the basal forebrain are attributed to the impairment of cognitive functions in Alzheimer’s disease (Perry et al., 1978; Bartus et al., 1982; Bartus, 2000). Later studies have challenged the hypothesis by showing that the selective cholinergic lesions in the basal forebrain do not induce memory deficits as expected by this hypothesis. Early stage Alzheimer’s patients do not show reduced cholinergic markers in the cortex (Davis et al., 1999; Dekosky et al., 2002). A few studies showed evidence of Alzheimer’s disease-related degeneration in the entorhinal cortex but not in the basal forebrain (Palmer, 2002; Pennanen et al., 2004). So the first neurodegenerative event in Alzheimer’s disease is possibly not the cholinergic depletion in the cortical structures (Gilmor et al., 1999; Dekosky et al., 2002; Mesulam, 2004). Some studies suggest that the degenerative process in the entorhinal cortex as the initial signs of Alzheimer’s disease (Gómez-Isla et al., 1996; Pennanen et al., 2004; Stoub et al., 2005) and this could lead to the cause of memory dysfunctions (Du et al., 2001; Rodrigue and Raz, 2004).

In the proposed model, the severity of AD is related to the percentage of neurons killed at the Central Layer, which represents the EC. The output of the network matches the behavioral response of AD patients at different levels of severity. The intact network produces the correct number-name responses for the given image input, which matches the responses of controls and AD patients at an early stage. The network with a lower percentage of neurons killed demonstrates some semantic error responses (correct number-type name response or wrong number-name of the same type), which matches the responses of mild-moderate stage of AD patients (ex. *tiger* instead of *lion* or *animal* instead of *lion*). A high percentage of neuronal loss shows semantic errors and no response (non-word response) that matches the response of the severe stage of AD patients (ex. *I don’t know*) (Barbarotto et al., 1998; Cuetos et al., 2005).

## Data availability statement

The original contributions presented in this study are included in the article/Supplementary material, further inquiries can be directed to the corresponding author.

## Author contributions

TK did simulations and wrote the main text. VSC contributed in providing the key ideas, editing the manuscript drafts, and providing insight into the model. BR contributed in providing key ideas and correcting the manuscript drafts. RM contributed in providing key ideas and correcting the manuscript drafts. All the authors contributed in the study concept and design.
