# Supplementary material for: A deep network-based model of hippocampal memory functions under normal and Alzheimer’s disease conditions
Source: Front Neural Circuits. 2023 Jun 21;17:1092933. doi: 10.3389/fncir.2023.1092933 (PMC10320296; doi:10.3389/fncir.2023.1092933)
Supplement: Supplementary file 1 [file Data_Sheet_1.PDF]

S1. Relating Alzheimer's patient's behaviour in picture naming Task

Figures S1-S3 relates three types of responses (correct response, semantic error, and no-response) demonstrated by AD participants in the picture naming task. Different stages of Alzheimer's disease (Controls, Mild-Moderate stage, and Severe stage) are imitated by inducing different percentages of neuronal loss (0%, 10%, and 50%), and the percentage of responses for each type is observed. Figure S1 shows the response percentage for the three groups when there is no neuronal loss. Here, a 100% correct response is observed and can be related to controls. Figure S2 shows the response percentages for 10% neuronal loss, where the correct response is higher, though not maximum, and some semantic error and no-response are observed, which can be related to the Mild-Moderate stage of AD patients. Figure S3 shows the responses for 50% neuronal loss, where the no-response is higher, and a higher number of semantic errors is also observed, which can be related to the severe stage of AD. Since a single character change in the actual word is also considered a non-word response, No-response is higher even at 10% neuronal loss.

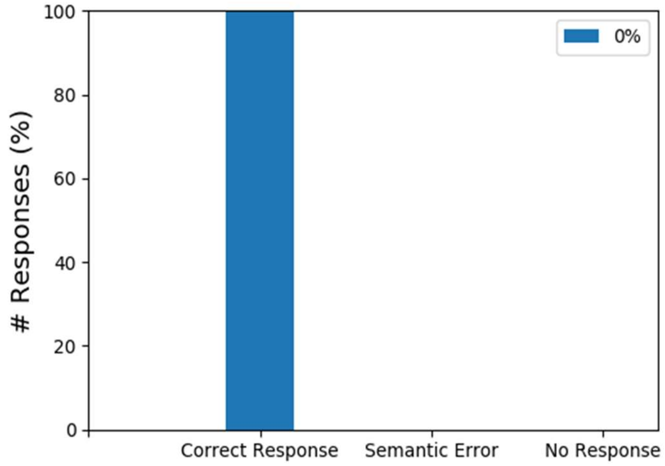

Figure S1 Control: when the neurons are intact (0% neuronal loss), 100% of correct response is observed.

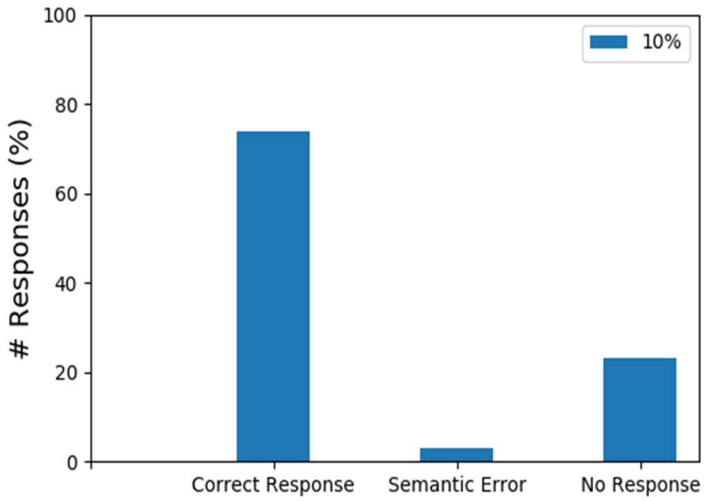

Figure S2 Mild to Moderate Stage: for 10% neuronal loss, more correct responses and some semantic errors are observed

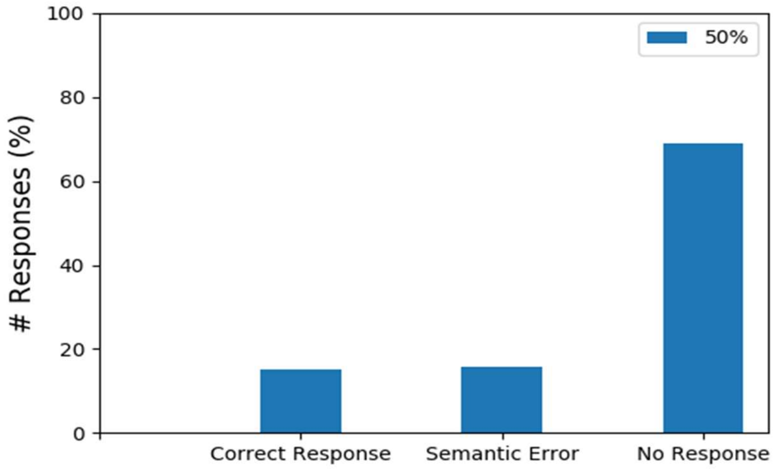

Figure S3 Sever Stage: for 50% neuronal loss, more of No response and some semantic errors are observed.

S2. Iteration Count to reach maximum familiarity value

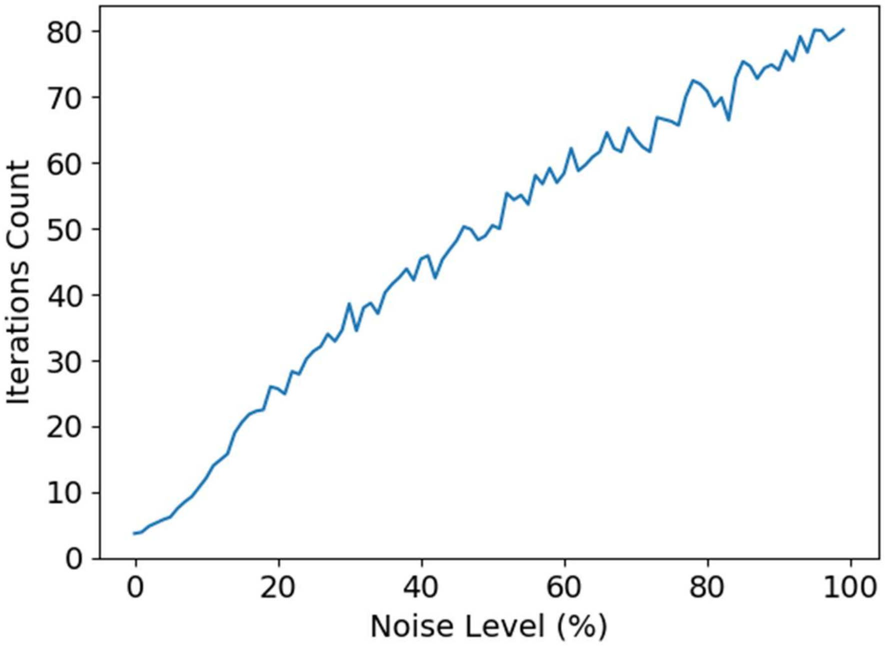

Figure S4 Number of iterations required to reach the maximum familiarity value vs. noise level.

We have counted the number of iterations required to reach the maximum familiarity value (.99) for different noise level (Figure S4). Here as the noise level increases the number of iterations also increases. It can be observed that the maximum value is attained in a finite number of iterations.
